# MiR-145 is upregulated in the retarded preimplantation embryos and modulates cholesterol levels in mice preimplantation embryos through targeting Abca1

**DOI:** 10.1186/s12958-022-01044-8

**Published:** 2022-12-12

**Authors:** Ou Jian, Ni MengXia, Xing Shiyu, Meng QingXia, Zou QinYan, Ding Jie, Wang Wei, Wan Jiaojiao, Li Hong, Huang Yining

**Affiliations:** 1 Center for Reproduction and Genetics, Suzhou Hospital of Nanjing Medical University, Suzhou Municipal Hospital, Suzhou, Jiangsu China; 2 Peking Jabrehoo Med-Tech Co., Ltd, No. 19 Tianrong Road, Daxing Bio-medicine Industry Park, Daxing District, Peking, 102629 China

**Keywords:** Preimplantation embryonic lethality, ICSI, miR-145, Abca1, Cholesterol

## Abstract

**Background:**

Preimplantation embryonic lethality is a driver of female infertility. Certain microRNAs (miRNAs) have previously been demonstrated to play important roles in the regulation of embryogenesis.

**Methods:**

Normally developing blastocysts and arrested embryos were collected from patients undergoing intracytoplasmic sperm injection (ICSI), and the expression of specific miRNAs therein was evaluated by qPCR. The overexpression of target molecule miR-145 in early mice embryos was achieved via oocyte microinjection, enabling the subsequent monitoring of how such overexpression impacted embryonic development. Bioinformatics approaches were utilized to identify putative miR-145 target mRNAs, and luciferase reporter assessments were implemented to confirm the ability of miR-145 to regulate Abca1 in HEK293T cells. The functional relationship between miR-145 and *Abca1* in the mice’s embryonic development was then confirmed through rescue assays.

**Results:**

Abnormally increased miR-145 expression was observed in patients’ arrested embryos, and the exogenous overexpression of this miRNA significantly suppressed mural blastocyst formation. Mechanistically, miR-145 was found to bind to the 3′-untranslated area of the Abca1 mRNA in HK293T cells, thus suppressing its expression and increasing embryonic cholesterol levels. In line with the importance of these cholesterol levels to embryogenesis, the upregulation of Abca1 was sufficient to rescue the observed change in cholesterol levels and the associated retardation of mice embryonic development that occurred in response to the overexpression of miR-145.

**Conclusion:**

The regulatory dynamics of the miR-145/Abca1 axis play an important role in shaping normal embryonic development.

## Background

Infertility affects 10-15% of couples and represents a growing clinical challenge worldwide [1]. The most common causes of infertility are ovulatory dysfunction, male factor infertility, and tubal disease. In addition, approximately 15% of infertile couples have “unexplained infertility” [2]. The emergence of assisted reproductive technology (ART) has proven to be of great value for couples suffering from infertility. Current live birth rates per ART treatment cycle are considerably higher when compared with the early years of ART, but overall, success rates remain low [3].

High rates of developmental arrest partly account for the observed low success rates, as only about 50% of embryos develop to the blastocyst stage despite improvements in culture media, laboratory equipment, and techniques [4]. Recent research derived from both embryonic studies and ART believes that preimplantation embryonic lethality (PEL) is a reason for initial female infertility [5]. In women with PEL, ovarian follicle expansion and ovulation usually proceed. Yet, women nonetheless suffer from recurrent in vitro fertilization (IVF) and intracytoplasmic sperm injection (ICSI) failures owing to a lack of successful fertilization or early embryonic arrest [6].

Dramatic changes ensure that a series of pivotal biological events proceed, including oocyte activation and maternal-to-zygotic transition coordinated with zygotic gene activation, followed by the first cell-fate decision and lineage-specific differentiation [7]. A precise regulatory network must function adequately to support such a significant shift in a short period. Epigenetic information is essential in maintaining cell characteristics and controlling gene expression. Epigenetic modifications, like DNA methylation, histone modifications, and noncoding RNA, have been found to play an irreplaceable role in the development of an embryo into a new individual [8].

MicroRNAs (miRNAs) are tiny (~ 22 nucleotides) non-coding RNAs that lack coding capability yet can control the permanence and expression of specific target mRNAs through binding to complementary succession within their 3′-untranslated region (UTR) domains. A great deal of data divulges that miRNAs are among the most abundant regulatory factors within cells, with individual miRNAs having the capability to control the expression of many target genes and individual genes similarly being subject to the regulatory activity of multiple miRNAs in some cases [9–13]. Both physiological and pathological activities have been attributed to miRNAs in human reproduction, and the dysregulated expression of many miRNAs has been noted in individuals suffering from specific reproductive disorders [14–16]. As such, there has been growing research interest in identifying and functionally characterizing those miRNAs influencing oocyte maturation and preimplantation embryonic development. For instance, Byrne et al. [17] proved that the loss of the miR-290-295 cluster in mice was associated with embryonic lethality due to the dysregulation of genes related to early embryonic development [18]. Consistently, the upregulation or downregulation of particular sets of miRNAs has been noted in many species in embryonic development, underscoring their dynamic regulatory importance in this developmental setting [16, 19–22].

To explain the mechanism of embryonic retardation caused by unknown factors, we attempted to identify some biomarkers that could influence embryonic development by comparing miRNA profiles of normally developing and arrested embryos. Here, we explored the expression of 12 different miRNAs in arrested and developing embryos and ultimately detected miR-145 as an aberrantly upregulated miRNA in arrested samples. We then explored the regulatory role of this miRNA via the microinjection of miR-145 mimics into murine oocytes and monitored the development and quality of the resultant embryos generated via IVF. We further identified *Abca1* as a putative miR-145 regulatory target and determined that miR-145 could suppress the protein level expression of ABCA1. Furthermore, we determined that ABCA1 overexpression could rescue mouse embryo arrest induced by miR-145 overexpression. These results suggest that miR-145\ABCA1 plays a significant role in the preimplantation development and could be used as a therapeutic target to improve the embryo quality.

## Materials and methods

### Informed consent

The Ethics Committee of the Suzhou Municipal Hospital confirmed this research (Research License 2,014,004). All embryos were obtained with written informed consent signed by the donor couples. The informed consent confirmed that the couple donors were voluntarily donating embryos for research on human early embryonic development mechanisms with no financial payment (Research License 2,014,004).

### Study subjects

From January 2018 to November 2019, 28 couples suffering from obstructive azoospermia (OA) were recruited from The Affiliated Suzhou Hospital of Nanjing Medical University. All of these couples met the following enrollment criteria: 1. Both partners were 20-40 years of age and men were diagnosed with OA; 2. The female partner did not exhibit any apparent abnormalities concerning the ovulatory status or oocyte morphology; 3. Table 1 details the clinical features of the 28 infertile women included in this paper; 4. The couples had undergone oocyte fertilization via ICSI. Spermatozoa (sperm cells) were obtained by microdissection from the testis or epididymis of male partners. All 28 couples have had successful pregnancies and births through ICSI at the center and have been authorized to remove excess embryos from freezing storage.

**Table 1:** Essential characteristics of the parental characteristics in this study

Parameters	Value
**Male**	
Age (years)	38.29 ± 5.49
BMI (kg/m2)	26.46 ± 1.98
**Female**	
Age (years)	36.94 ± 6.48
BMI (kg/m^2^)	22.64 ± 2.62
Duration of infertility (years)	6.98 ± 4.37
AMH (ng/ml)	1.09 ± 0.94
FSH(mIU/mL)	11.02 ± 3.57
Estradiol (pg/ml)	190.46 ± 148.62
Antral follicle count	3.02 ± 1.49

### Collection and culture of human embryos

Each couple donated one or two cryopreserved Day 3 cleavage-stage embryos, a total of 51. The embryos were thawed rapidly by taking straws from the liquid nitrogen storage tank, exposing them to air for 40 s, and immersing them in a water bath at 30 °C for 1 min. Embryos were sequentially placed into thawing medium drops for 5 min with decreasing PROH concentrations (1.0 mol/l, then 0.5 mol/l and finally 0 mol/l), each with 0.2 mol/l sucrose at room temperature to remove the cryoprotectant. Thawed embryos were transferred to PBS with 20% HSA for 10 min at 37 °C and then to G2 culture medium (Vitrolife) to evaluate blastomere survival.

The G-2 (Vitrolife) medium was used to culture the 8-cell embryos to the blastocyst stage. At the 6th day, the blastocysts with a thin zona pellucida, smooth trophectoderm, clearly visible blastocyst cavity and well-developed inner cell mass were collected as “Normal Controls”. The arrested 8-cell and morulae embryos that have no signs of degeneration were collected as “Arrested embryo”. In this study, 15 normal controls and 15 arrested embryos were collected for the miRNA quantification.

### Embryonic phenotype analyses

The morphological characteristics of embryos during different stages of development were assessed via light microscopy (IX-71, Olympus, Japan). Embryo quality was assessed at pre-selected time points using standard guidelines [23].

### Mice

Wild-type (WT) ICR mice were acquired from the Laboratory Animal Center of Nanjing Medical University, China. They were kept in specific pathogen-free circumstances in a climate-controlled setting (50-70% humidity, 20–22 °C, 12 h light/dark cycle) with free access to water and food. Animal experiments were approved (license number 2004020) by the Animal Ethical and Welfare Committee of Nanjing Medical University (Nanjing, China).

### Superovulation and oocyte collection

Female 21–23 days old mice were intraperitoneally injected with 5 IU of pregnant mare’s serum gonado¬tropin (PMSG, Ningbo Sansheng Pharmaceutical) to induce superovulation. At 46 h post-injection, mice were euthanized by cervical dislocationThe full grown oocytes (FGO) were collected by manual puncture of ovarian follicles and maintained in M2 medium (Sigma, St. Louis, MO, USA) containing 2 μM milrinone to inhibit GVBD.

### Oocyte microinjection

The overexpression of miR-145 and miR-145-mu were attained through purchasing miR-145 mimic\mu from GenePharma and diluting them to a final concentration of 20 uM. To overexpress *Abca1*, a capped cRNA was synthesized as described below. An Eppendorf Transferman NK2 micromanipulator was utilized to conduct the microinjection of the miRNA mimic\mu,cRNA or distilled water (Control), to denuded oocytes with an injection volume of 5-10 pL. In addition, a group of embryos that had not undergone microinjection served as culture controls (Normal). Once this injection was complete, oocytes were rinsed and cultured in an M16 medium for 14 h.

### In vitro fertilization

A moderately revised version of an already reported approach was used to conduct all murine IVF experiments [24]. Sperm were collected from dissected epididymis samples from 10 to 12 week-old ICR mice. Following the incubation of these samples in HTF medium (Millipore, Merck) containing 10 mg/ml BSA for 1 h, dispersed spermatozoa were added to HTF drops with oocytes. Following coincubation at 37 °C in an incubator for 4-6 h, the presumptive zygotes were rinsed and transferred to KSOM media (Millipore). They were cultured to the blastocyst stage at 37 °C in a moisturized incubator under a 5% CO2, 5% O2, and 90% N2 atmosphere.

### Cell culture

HEK293T cells were procured from the China Infrastructure of Cell Line Resources and grown in DMEM supplied with 10% fetal bovine serum (FBS; Hyclone, UT, USA) and penicillin/streptomycin. The cells were cultured in a 37 °C 5% CO2 incubator.

### qPCR

Trizol (Invitrogen, CA, USA; 15,596,026) was used based on the presented directions to extract cellular RNA (5 embryos were pooled), followed a TaqMan miRNA RT-Real Time PCR was employed for the detection of miRNA expression. Briefly, a TaqMan MicroRNA Reverse Transcription Kit (Applied Biosystems, CA, USA; 4,366,596) was used to prepare cDNA, followed by amplification with TaqMan Universal PCR Master Mix (Applied Biosystems, CA, USA; 4,304,437) and TaqMan™ MicroRNA Assay (Applied Biosystems, CA, USA; 4,440,885). Normalization was conducted using U6 as a control. Samples were analyzed in triplicate, with a minimum of three repeats per experiment.

### Dual-luciferase reporter assay

A 956 bp segment of the Abca1 3′-UTR comprising the predicted miR-145 binding site was cloned into the pmirGLO plasmid (Promega, WI, USA; FJ376737) upstream of the firefly luciferase gene. HEK293T cells were seeded in the plates containing 48 wells and transfected by implementing 20 nM of the appropriate miRNA mimic or control constructs employing Lipofectamine 2000 (Invitrogen, CA, USA; 11,668,019) along with the prepared luciferase reporter vector (200 ng/well). 48 h later, cells were harvested and analyzed by applying a Dual-Luciferase Assay kit (Promega, WI, USA; E1910). The analyses were executed in triplicate, and assessments were repeated three times. Firefly luciferase activity was normalized to Renilla luciferase activity for downstream analysis.

### Western blotting

Samples were incubated at 95 °C for 5 min in 2 × SDS sample buffer and stored at − 20 °C. For the embryos, a total of 100 samples per group were studied. For 293 T Cells, 5 × 10^6^ cells per group were used. Proteins extracts were separated using 10% SDS-PAGE and transfer onto PVDF membranes (Amersham Pharmacia Biotech, Herts, UK). Blots were then proved overnight with monoclonal mouse anti-ABCA1 (Abcam, MA, USA; ab18180) or mouse anti-GAPDH (Abcam, MA, USA; ab8245) at 4 °C. Appropriate HRP-conjugated anti-mouse IgG secondary antibodies were then used for protein detection, followed by an ECL kit (Thermo Scientific™, 32,106) was implemented to visualize protein bands. GAPDH was employed as a loading control.

### Cholesterol analyses

Total embryonic lipid content was extracted using methanol-chloroform, followed by a cholesterol determination kit (Sigma-Aldrich, MO, USA; MAK043) based on provided directions to measure cholesterol content.

### In vitro transcription of cRNA

Murine *Abca1* plasmids were purchased from GeneCopoeia. Murine *Abca1* was amplified from murine embryonic cDNA and cloned into the pCS2+ vector, which harbors a Myc tag, enabling in vitro polyadenylated mRNA transcription [25]. SacII or KpnI were used for construct linearization, followed by gel extraction kit (Promega, WI, USA; A9281)-mediated purification. Capped mRNAs were generated with an SP6 message machine (Ambion, CA, USA; AM1340) employed for generating capped mRNAs, and a RNeasy cleanup kit (Qiagen, 74,204) was utilized for subsequent mRNA purification.

### Statistical analysis

**Fig. 1:**
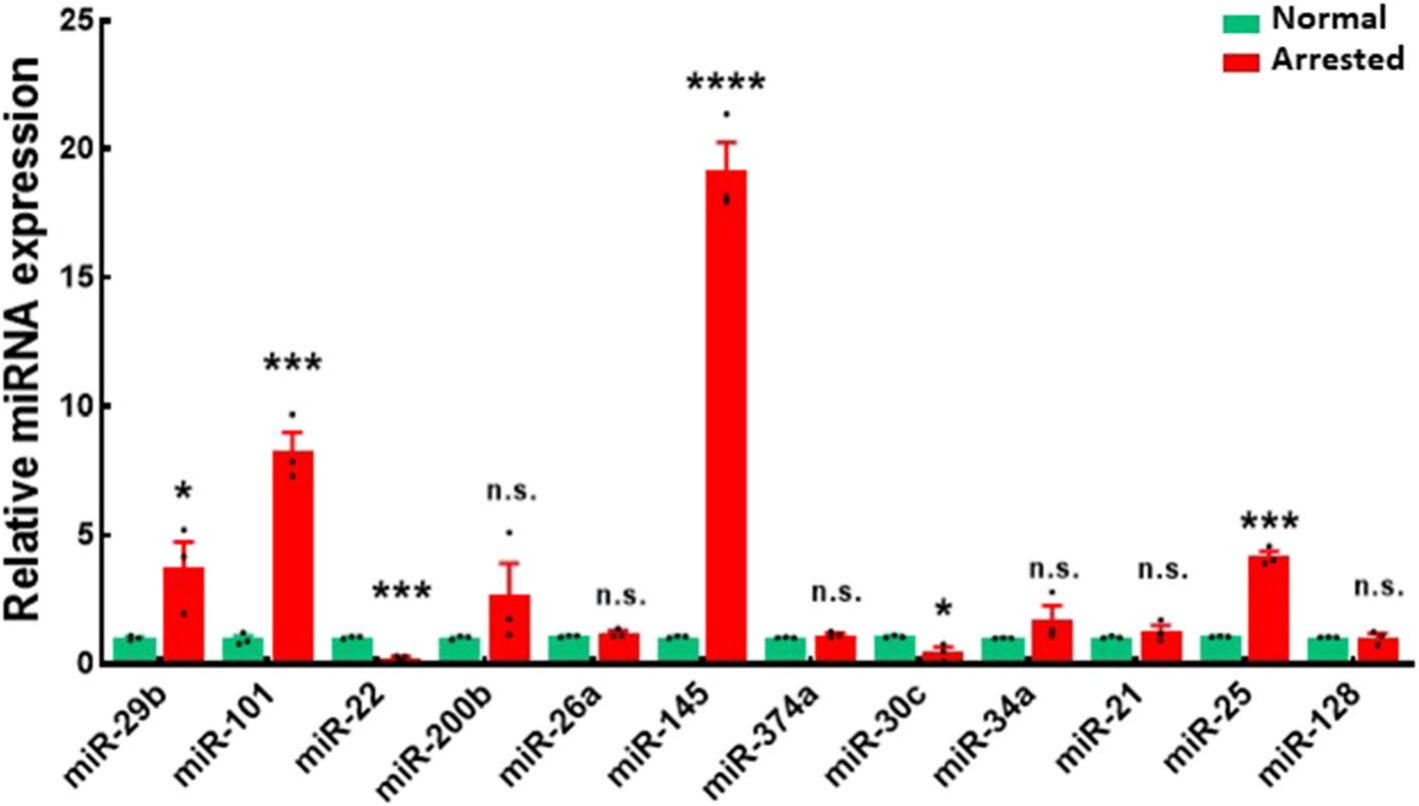
miR-145-5p is upregulated in arrested embryos. Different miRNAs’ expression was assessed in arrested embryos and normal controls via qPCR. Five embryos were pooled together as a group. Data are presented as means ± s.d. representing 3 biological replicates. Total number of embryos analyzed per group: *n* = 15

**Fig. 2:**
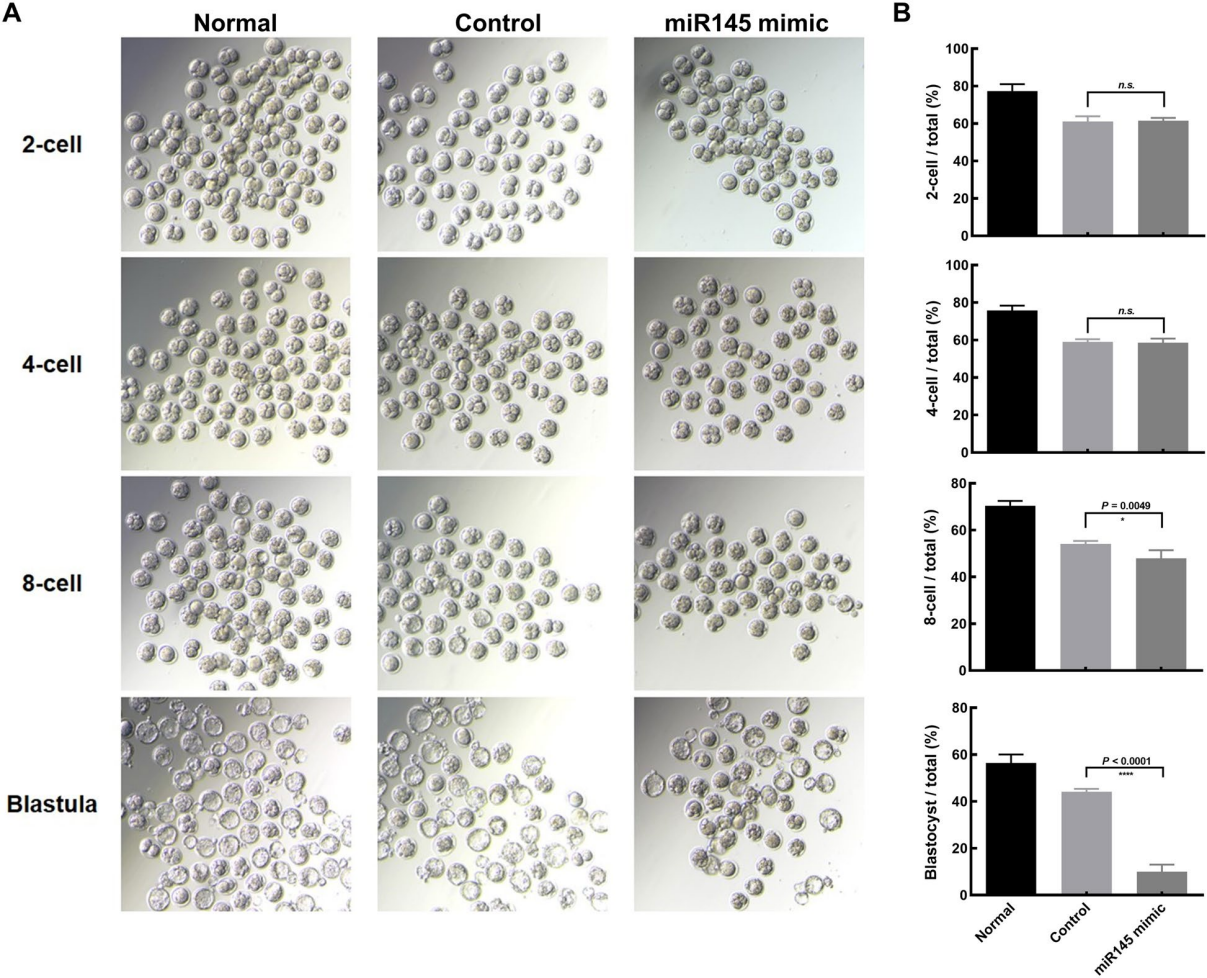
miR-145 suppresses murine embryogenesis. Morphological (**A**) and quantitative (**B**) analyses of the 2-cell, 4-cell, 8-cell, and blastocyst stages of murine oocyte advancement following miR-145 mimic (*n* = 126) or negative control (Control) (*n* = 102) microinjection into oocytes, with untreated control samples (Normal) (*n* = 189) being analyzed in parallel. The images were taken at 16 h (2-cell), 40 h (4-cell), 52 h (8-cell), and 74 h (blastocyst) after transfer of the zygotes to KSOM. The assessments have been repeated five times. ANOVA test, **P < 0.01; ****P < 0.0001. NS, no significance

**Fig. 3:**
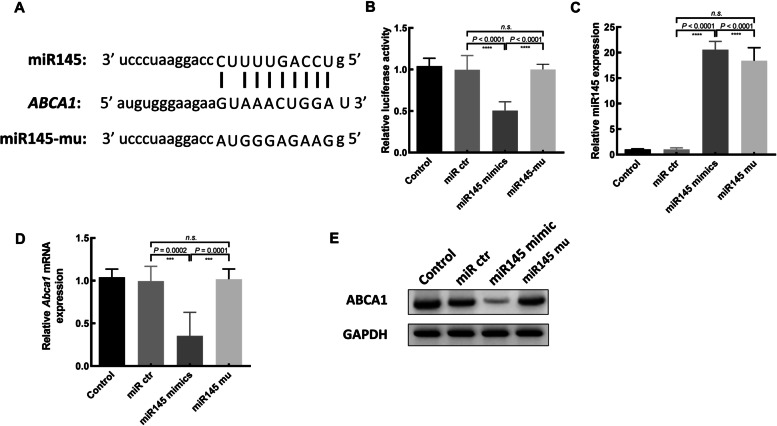
miR-145 directly targets Abca1. **A** Putative sequence complementarity between miR-145 and Abca1. **B** miR-145 mimic transfection was adequate to suppress the luciferase activity of a reporter containing the Abca1 3′-UTR segment harboring the binding site shown in (A) (*n* = 3). **C** The overexpression of miR-145 in 293 T cells was confirmed via qPCR. **D**, **E** Abca1 mRNA and protein levels (cropped gels/blots) were assessed by means of qPCR and Western blotting after the indicated treatments. All the data are means ± s.d. from five independent experiments

**Fig. 4:**
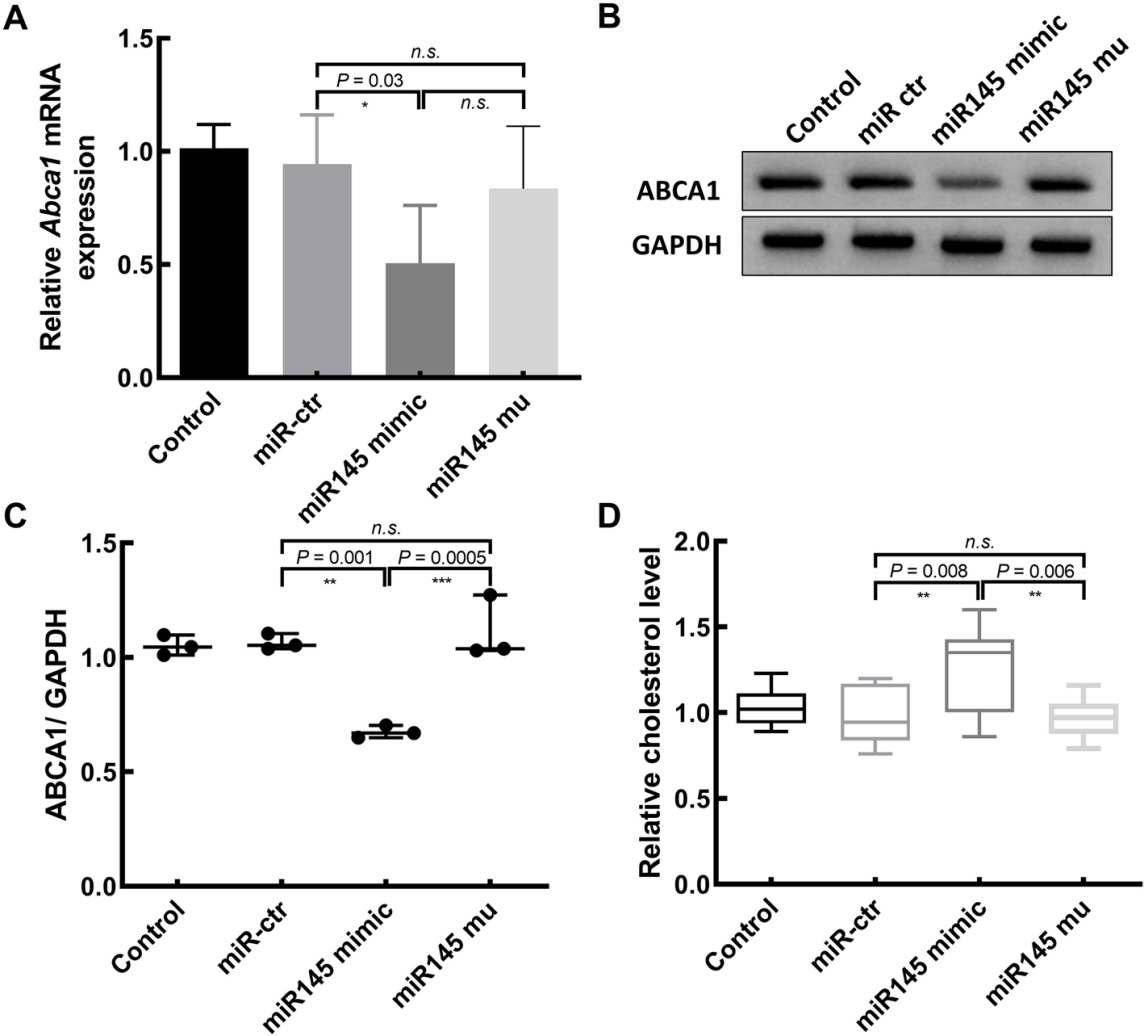
miR-145 inhibits Abca1 expression and thereby increases cholesterol levels within embryos. **A**, **B** Abca1 expression at the mRNA level in blastocysts was assessed following overexpression of miR-145 in oocytes. Data are presented as means ± s.d. from five independent experiments (total number of blastocysts analyzed: *n* = 241 for control, 239 for miR-ctr, 226 for miR145 mimic and 229 for miR145 mu). **B**, **C** Representative western blot analysis for ABCA1 accumulation in blastocysts obtained from each groups (100 embryos per lane). One representative of three independent experiments is shown (B). Data are shown as medians with ranges. **D** miR-145 was associated with significantly increased cholesterol levels. Data are presented as means ± s.d. from five independent experiments (total number of *n* = 228 for control, *n* = 222 for miR-ctr, *n* = 212 for miR145 mimic and *n* = 218 for miR145 mu)

**Fig. 5:**
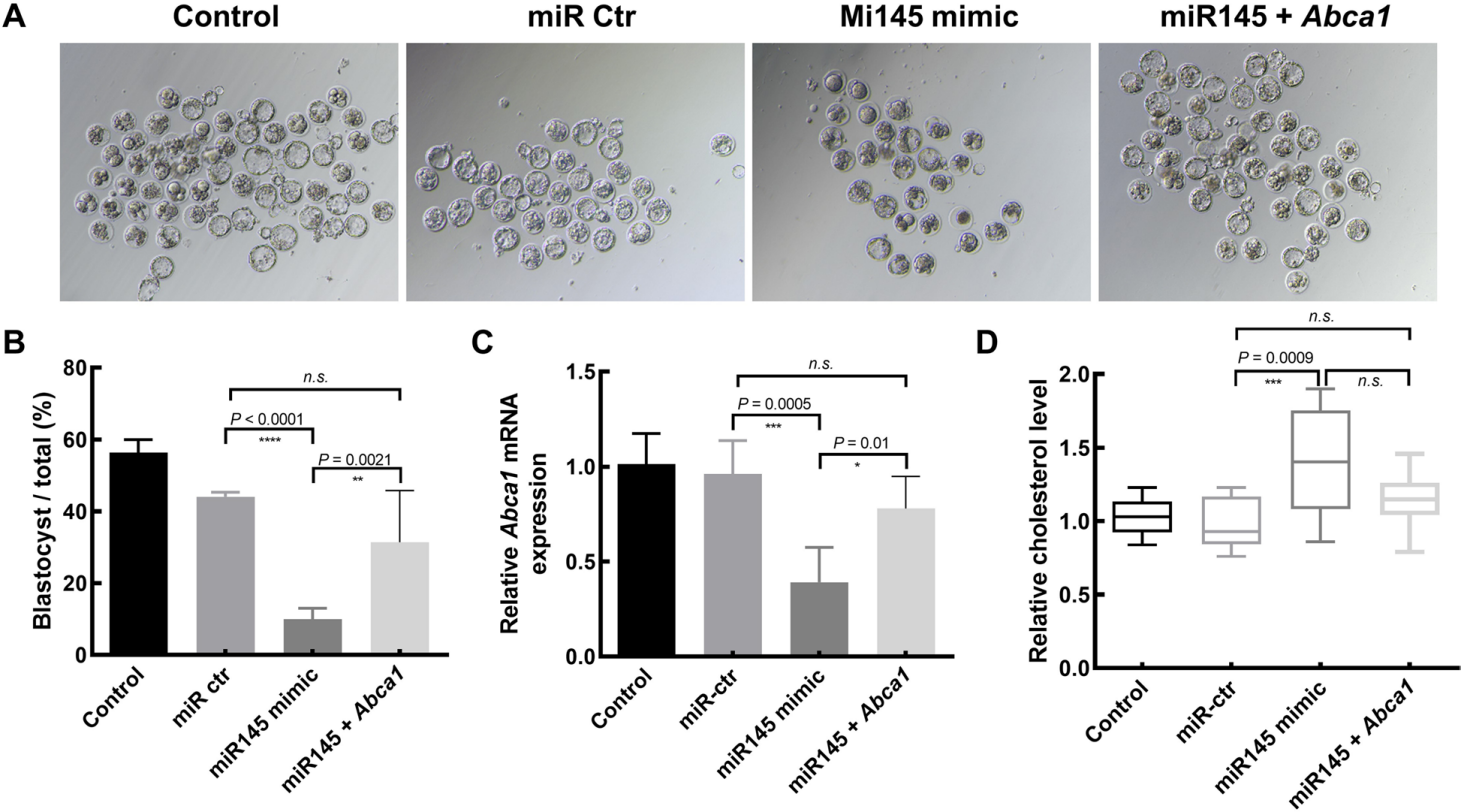
miR-145 regulates Abca1 expression to control murine embryonic development. **A**, **B** The overexpression of Abca1 was sufficient to reverse the retardation of embryonic growth observed in response to miR-145 overexpression. The images of embryos were taken at 74 h after transfer of the zygotes to KSOM. **C** Abca1 expression in the indicated groups was assessed via qPCR. Total number of zygotes used in (A)-(C), *n* = 180 for control, 121 for miR-ctr, 115 for miR145 mimic and 97 for miR145 + Abca1. Data are presented as means ± s.d. from five independent experiments. **D** Abca1 overexpression was sufficient to reverse miR-145 overexpression-induced increases in embryonic cholesterol levels. Total number of zygotes used in (D), *n* = 232 for control, 218 for miR-ctr, 209 for miR145 mimic and 195 for miR145 + Abca1. Data are presented as means ± s.d. from five independent experiments

The statistical assessments were conducted by implementing GraphPad Prism 9.0 (GraphPad Inc., CA, USA). Statistical comparison associated with Fig. 1 was Student’s t-tests; Statistical comparison used in Figs. 2, 3, 4A, D and Fig. 5 were ANOVA test (followed by the Tukey Honestly Significant test); Statistical comparison used in Fig. 4C was Kruskal-Wallis followed by Dunns post hoc test. *P* < 0.05 was the significance threshold (*P < 0.05; **P < 0.01; ***P < 0.001; ****P < 0.0001).

## Results

### Analysis of differential miRNA expression patterns in arrested blastocysts

We used donated embryos to detect the expression patterns of 12 different miRNAs.. To exclude obvious maternal factors, we selected patients whose disease was due to male OA, and all examination parameters of females were normal, as described in detail in the materials and methods.

Through qPCR, miR-29b, miR-101, miR-200b, miR-145, miR-34a, and miR-25 were found to be upregulated in arrested blastocysts (*P* < 0.05). At the same time, miR-22 and miR-30c were downregulated relative to normal control embryos (*P* < 0.05). No differences in miR-26a, miR-374a, miR-21, or miR-128 expression were observed between these groups (Fig. 1). Given that miR-145 exhibited the highest levels of differential expression among the surveyed miRNAs, we hypothesized that it could perform an essential task in the context of embryonic development.

### miR-145 overexpression in murine oocytes modulates early embryonic potential

To discover the effective functional task of miR-145 during the early stages of embryogenesis, we used a miR-145 mimic microinjection technique to overexpress it in murine oocytes. These oocytes were then utilized for IVF assays conducted using normal ICR mouse sperm, with the rates of subsequent embryonic development monitored. By this approach, we found that normal, control, and miR-145-overexpressing embryos all developed normally beyond the two-cell stage (normal, 77.4%; control, 61.16%; miR-145, 61.42%) and into the four-cell stage (normal, 75.8%; control, 58.92%; miR-145, 58.62%) (Fig. 2). However, a significant reduction in the proportion of 8-cell embryos were observed in the context of miR-145 overexpression (normal, 70.4%; control, 54.12%; miR-145, 47.98%), and most of miR-145 overexpressed embryos could not develop into the blastula (normal, 56.4%; control, 44.12%; miR-145, 9.98%) (Fig. 2). These data suggest that overexpressing miR-145 can result in the arrest of embryonic development.

### miR-145 directly targets Abca1 to regulate its expression

To further illuminate the regulatory roles of miR-145 in the context of embryogenesis, we used the Targetscan database (www.targetscan.org) to identify putative miR-145 target genes. Revealing Abca1 is one such target gene (Fig. 3A). To confirm the capability of miR-145 for direct regulation of the expression of murine *Abca1*, we generated a luciferase reporter construct harboring the *Abca1* 3′-UTR sequence predicted to harbor a miR-145 binding site. A dual-luciferase reporter assessment was then conducted. And the reduction in relative luciferase activity was detected in cells transfected with miR-145 (Fig. 3B), while this effect was abolished when 9 nucleotides within the putative miR-145 binding region were mutated.

To clarify whether miR-145 can regulate *ABCA1* expression under endogenous conditions, the 293 T cells were transfected with miR-145 mimic or mutant control constructs. The expression of miR-145 and ABCA1 at the protein and mRNA levels were assessed. As expected, *ABCA1* mRNA and protein levels were substantially reduced in cells transfected with the miR-145 mimic but not the mutant version of this construct (Fig. 3C-E). It was revealed that Abca1 is a target gene.

### miR-145 influences embryonic cholesterol levels via the regulation of ABCA1 protein expression

To fully clarify the functional role of embryonic miR-145, we microinjected murine oocytes with miR-145 or a mutant control construct. These oocytes were then used to prepare blastocysts via IVF, and *Abca1* expression was appraised at the protein and mRNA levels through Western blotting and qPCR assays. This approach revealed that ABCA1 was significantly downregulated at the protein and mRNA levels in miR-145 overexpression, whereas the mutant miRNA construct failed to impact such expression (Fig. 4A-C).

ABCA1 is a crucial regulator of phospholipid and cholesterol homeostasis in cells that are also referred to as CERP (cholesterol efflux regulatory protein). Abnormal ABCA1 expression patterns can lead to the aberrant accumulation or depletion of intracellular cholesterol. We thus assessed cholesterol levels in the embryos in our different treatment groups, revealing a significant increase in cholesterol levels in embryos overexpressing miR-145 (Fig. 4D).

### Appropriate miR-145/Abca1 homeostasis is essential for normal embryonic development

To directly evaluate the mechanistic link between miR-145 upregulation and embryonic developmental arrest, we overexpressed *Abca1* in oocytes overexpressing miR-145 using the same microinjection technique. We then compared the embryo to blastocyst ratio in each group. This analysis revealed that the overexpression of miR-145 significantly decreased embryo competence, as evidenced by reduced blastocyst development (Fig. 5A, B), consistent with our findings above. However, the overexpression of *Abca1* was sufficient to partially rescue such miR-145-mediated inhibition of embryonic development (Fig. 5A, B). As expected, *Abca1* mRNA levels were lower in embryos that had been microinjected with miR-145. At the same time, this was reversed in the context of supplemental *Abca1* mRNA microinjection (Fig. 5C). In addition, *Abca1* microinjection was sufficient to reverse the miR-145-induced increase in cholesterol levels within these embryos (Fig. 5D). Together, these data thus suggest that miR-145 can impair embryonic development. In contrast, the *Abca1*-mediated restoration of cholesterol homeostasis can restore normal embryogenesis.

## Discussion

Many couples affected by infertility experience recurrent IVF/ICSI failure. In some cases, patients may exhibit normal ovulatory status and morphologically normal oocytes yet are nonetheless affected by severely impaired zygote formation and embryonic development. The failed fertilization and embryonic arrest phenotypes observed in these patients are collectively referred to as PEL. Herein, we sought to discover the molecular determinants of such fertility outcomes by collecting normal and arrested embryos from couples undergoing ICSI treatment at our center. When we scrutinized the expression levels of different miRNAs in these embryos via qPCR, we found that miR-145 was overexpressed in the arrested embryos relative to the normally developing embryos, suggesting high levels of expression of this miRNA may be tied to impaired embryonic development. Furthermore, mouse embryo experiments demonstrated that the high level of miR145 inhibited embryonic development, especially the development from 8-cells to blastocyst stage. Therefore, our data suggest that miR145 plays a regulatory role during preimplantation embryonic development.

Similar miRNA profiles in both embryos during the early stages of development and mature murine oocytes suggest that zygotes primarily contain maternally-derived miRNAs [26]. The expression of these miRNAs is reduced by up to 60% between the 1- and 2-cell stages of development but doubles at the end of the 4-cell stage relative to the 2-cell stage, consistent with embryonic genomic activation initiating somewhere between the 1- and 4-cell stages of embryogenesis [26]. While miRNAs are routinely synthesized and degraded throughout murine preimplantation embryo development, an overall rise in miRNA expression levels is observed towards the blastocyst stage [27]. While many studies have examined miRNA expression dynamics in mice, the specific functional tasks of individual miRNAs in the context of early embryonic development remain poorly understood [28, 29].

miR-145 has been reported to repress the expression of genes associated with pluripotency in human ES cells in prior studies [30]. And treatment of nuclear-transferred embryos with a miR-145 inhibitor improves developmental competence and quality. miR-145 was chosen as a target for additional research. Other research suggests that miR-145 can target a range of oncogenes, thereby modulating cellular proliferative, migratory, invasive, and apoptotic activity [31]. When we employed a microinjection approach to overexpress miR-145 in murine oocytes, we found that such overexpression significantly increased the rate of arrest during embryogenesis, suggesting that high levels of miR-145 expression can trigger arrest during embryonic development.

ABCA1 is an ABC1 superfamily member responsible for transporting cholesterol and phospholipids across the cell membrane to HDL-C, thus playing a key role in regulating cholesterol homeostasis, endothelial function, blood pressure regulation, vascular inflammation, and platelet aggregation in the context of atherosclerotic vascular disease [32]. Several miRNAs have been reported to regulate *Abca1* expression, including miR-33a, miR-122, miR-467b, miR-183, and miR-28 [33, 34]. miR-145 has been found to promote hypoxia-induced proliferation and migration of pulmonary arterial smooth muscle cells by regulating ABCA1 expression [35]. However, so far, no studies have investigated the function of ABCA1 during preimplantation embryo development. Here, we verified that *Abca1* is a straightforward miR-145 target in mice embryos, with miR-145 overexpression consequently resulting in significant decrease in ABCA1 levels within embryos, potentially contributing to the consequent incidence of developmental arrest during embryogenesis.

Cholesterol has been found to be essential for preimplantation embryo development, and cholesterol depletion inhibited preimplantation development [36]. Cholesterol is one of the main components of lipid rafts. Mouse preimplantation embryos express stage-dependent distribution of lipid rafts, which possibly play crucial roles in cytokinesis and cell signaling events at this stage by acting as a signal organizing platform [36]. In addition, Pawlak et al. identified significant involvement of cholesterol metabolism in preimplantation embryos by RNA sequencing of porcine and bovine preimplantation embryos [37]. In our study, we found that *Abca1* overexpression in this experimental system was sufficient to reverse the miR-145-induced arrest of embryogenesis through mechanisms tied to reductions in cholesterol content within embryos. This data suggests that too much cholesterol can also arrest the development of preimplantation embryos. Therefore, the maintenance of cholesterol homeostasis is the prerequisite to ensure the normal development of preimplantation embryos. Further studies on the function of cholesterol in embryos will also benefit the advancement of assisted reproductive technology.

## Conclusions

Our study elucidated the function of miR145 in preimplantation embryo development and suggests that miR145 may be a clinical target for improving embryo development rate. miR145 regulates the expression of *Abca1* in preimplantation embryos by binding to the 3′ non-coding region of *Abca1*, a cholesterol transport protein, and thus maintains cholesterol homeostasis in the embryo, which is an important guarantee for the normal development of the embryo.

## Data Availability

The results/data/figures in this manuscript have not been published elsewhere, nor are they under consideration (from me or one of any Contributing Authors) by another publisher. All of the material is owned by the authors and/or no permissions are required.

## Abbreviations

ART: Assisted reproductive technology
BSA: Bismuth Sulphite Agar
CERP: Cholesterol efflux regulatory protein
DMSO: Dimethylsulfoxide
FBS: Fetal bovine serum
GV: Germinal vesicle
hCG: Human chorionic gonadotropin
HTF: Human tubal fluid
ICR mouse: Institute-for-cancer-research-mouse
ICSI: Intracytoplasmic sperm injection
IVF: In vitro fertilization
KSOM: Potassium Simplex Optimzed Medium
OA: Obstructive azoospermia
PEL: Preimplantation embryonic lethality
PMSG: Pregnant mare’s serum gonado¬tropin
UTR: Untranslated region
WT: Wild-type

## References

[1] Tamrakar S, Bastakoti R (2019). Determinants of infertility in couples. J Nepal Health Res Counc.

[2] Carson S, Kallen A (2021). Diagnosis and management of infertility: a review. JAMA.

[3] Rødgaard T, Heegaard P, Callesen H (2015). Non-invasive assessment of in-vitro embryo quality to improve transfer success. Reprod BioMed Online.

[4] McCollin A (2020). Abnormal cleavage and developmental arrest of human preimplantation embryos in vitro. Eur J Med Genet.

[5] Yatsenko S, Rajkovic A (2019). Genetics of human female infertility†. Biol Reprod.

[6] Zhang M (2021). TLE6Identification of novel biallelic variants in female infertility with preimplantation embryonic lethality. Front Genet.

[7] Jukam D, Shariati S, Skotheim J (2017). Zygotic genome activation in vertebrates. Dev Cell.

[8] Xu R (2021). Insights into epigenetic patterns in mammalian early embryos. Protein Cell.

[9] Bartel D (2004). MicroRNAs: genomics, biogenesis, mechanism, and function. Cell.

[10] Abrahante J (2003). The Caenorhabditis elegans hunchback-like gene lin-57/hbl-1 controls developmental time and is regulated by microRNAs. Dev Cell.

[11] Gottesman S (2004). The small RNA regulators of Escherichia coli: roles and mechanisms*. Annu Rev Microbiol.

[12] Lewis B, Burge C, Bartel D (2005). Conserved seed pairing, often flanked by adenosines, indicates that thousands of human genes are microRNA targets. Cell.

[13] Wienholds E (2005). MicroRNA expression in zebrafish embryonic development. Science (New York, NY).

[14] Hossain MM (2009). Identification and characterization of miRNAs expressed in the bovine ovary. BMC Genomics.

[15] Tesfaye D (2009). Identification and expression profiling of microRNAs during bovine oocyte maturation using heterologous approach. Mol Reprod Dev.

[16] Abd El Naby WS (2013). Expression analysis of regulatory microRNAs in bovine cumulus oocyte complex and preimplantation embryos. Zygote.

[17] Byrne MJ, Warner CM (2008). MicroRNA expression in preimplantation mouse embryos from Ped gene positive compared to Ped gene negative mice. J Assist Reprod Genet.

[18] Medeiros LA (2011). Mir-290-295 deficiency in mice results in partially penetrant embryonic lethality and germ cell defects. Proc Natl Acad Sci U S A.

[19] Giraldez AJ (2006). Zebrafish MiR-430 promotes deadenylation and clearance of maternal mRNAs. Science.

[20] Mineno J (2006). The expression profile of microRNAs in mouse embryos. Nucleic Acids Res.

[21] Gilchrist GC (2016). MicroRNA expression during bovine oocyte maturation and fertilization. Int J Mol Sci.

[22] Goossens K (2013). Regulatory microRNA network identification in bovine blastocyst development. Stem Cells Dev.

[23] Ding D, et al. Effects of different polyvinylpyrrolidone concentrations on intracytoplasmic sperm injection. Zygote. 2020:1–6. 10.1017/S0967199419000820. 10.1017/S0967199419000820 31933453

[24] Plück A, Klasen C (2009). Generation of chimeras by microinjection. Methods Mol Biol.

[25] Wang Q (2008). Down-regulation of Sonic hedgehog signaling pathway activity is involved in 5-fluorouracil-induced apoptosis and motility inhibition in Hep3B cells. Acta Biochim Biophys Sin Shanghai.

[26] Tang F (2007). Maternal microRNAs are essential for mouse zygotic development. Genes Dev.

[27] Yang Y (2008). Determination of microRNAs in mouse preimplantation embryos by microarray. Dev Dyn.

[28] McCallie B, Schoolcraft WB, Katz-Jaffe MG (2010). Aberration of blastocyst microRNA expression is associated with human infertility. Fertil Steril.

[29] Rosenbluth EM (2013). MicroRNA expression in the human blastocyst. Fertil Steril.

[30] Xu N (2009). MicroRNA-145 regulates OCT4, SOX2, and KLF4 and represses pluripotency in human embryonic stem cells. Cell.

[31] Cui SY, Wang R, Chen LB (2014). MicroRNA-145: a potent tumour suppressor that regulates multiple cellular pathways. J Cell Mol Med.

[32] Schumacher T, Benndorf RA. ABC Transport Proteins in Cardiovascular Disease-A Brief Summary. Molecules. 2017;22(4):589. 10.3390/molecules22040589. 10.3390/molecules22040589 PMC6154303 28383515

[33] Rotllan N (2016). microRNAs in lipoprotein metabolism and cardiometabolic disorders. Atherosclerosis.

[34] Yang Z, Cappello T, Wang L (2015). Emerging role of microRNAs in lipid metabolism. Acta Pharm Sin B.

[35] Yue Y (2018). miR-143 and miR-145 promote hypoxia-induced proliferation and migration of pulmonary arterial smooth muscle cells through regulating ABCA1 expression. Cardiovasc Pathol.

[36] Comiskey M, Warner CM (2007). Spatio-temporal localization of membrane lipid rafts in mouse oocytes and cleaving preimplantation embryos. Dev Biol.

[37] Kajdasz A, Warzych E, Derebecka N, et al. Lipid Stores and Lipid Metabolism Associated Gene Expression in Porcine and Bovine Parthenogenetic Embryos Revealed by Fluorescent Staining and RNA-seq. Int J Mol Sci. 2020;21(18):6488. 10.3390/ijms21186488. 10.3390/ijms21186488 PMC7555686 32899450

